# The independent and joint associations of community space environmental satisfaction, and parents’ educational attainment with psychological symptoms among Chinese adolescents

**DOI:** 10.3389/fpubh.2026.1856733

**Published:** 2026-07-15

**Authors:** Cunbi Bi, Qin Yang, Feng Zhang, Jian Dong

**Affiliations:** 1 School of Design, Zhejiang Vocational Academy of Art, Hangzhou, Zhejiang, China; 2 School of Physical Education, Chizhou University, Chizhou, China; 3 School of Sports Science and Engineering, East China University of Science and Technology, Shanghai, China; 4 School of Physical Education, Anqing Normal University, Anqing, China

**Keywords:** adolescents, community space environment, cross-sectional study, parents’ educational attainment, psychological symptoms

## Abstract

**Background:**

The prevalence of psychological symptoms among Chinese adolescents remains high, negatively impacting their academic performance and future achievements. Previous research has paid relatively little attention to the association between community space environment and parents’ educational attainment and psychological symptoms. This study may provide valuable insights and guidance for the prevention and intervention of psychological symptoms among Chinese adolescents.

**Methods:**

Using a stratified cluster sampling across six regions of China to assess the community spatial environment, parents’ educational attainment, and psychological symptoms. Chi-square tests, *t*-tests, and binary logistic regression were used to analyze the associations between the community spatial environment and parents’ educational attainment and psychological symptoms.

**Results:**

The prevalence of psychological symptoms among Chinese adolescents is 21.2%. The rate is higher among boys (22.1%) than among girls (20.4%), and this difference is statistically significant (*χ*^2^ = 21.055, *p* < 0.001). Binary logistic regression analysis showed that, with adolescents in the group reporting “Satisfied” with community space environmental satisfaction and “University degree or above” for parents’ educational attainment serving as the reference group, adolescents in the group reporting “Not satisfied” with community space environmental satisfaction and “Junior high school and below” for parents’ educational attainment had the highest risk of developing psychological symptoms (OR = 2.04, 95% CI: 1.81–2.30) (*p* < 0.001).

**Conclusion:**

The prevalence of psychological symptoms among Chinese adolescents is relatively high, with boys exhibiting a higher prevalence than girls. There are independent and joint associations between satisfaction with the community environment and both parents’ educational attainment and adolescents’ psychological symptoms. Improving the community environment and increasing parents’ educational attainment may reducing psychological symptoms among adolescents.

## 1 Introduction

As living environments and lifestyles continue to change, the prevalence of psychological symptoms among adolescents has been steadily rising, becoming a major public health issue faced by countries around the world (1). A survey of U.S. adolescents shows that between 2018 and 2022, the prevalence of psychological symptoms among U.S. adolescents rose from 27.8 to 35% (2). China is no exception. Survey data from 2005 to 2011 show that the prevalence of psychological symptoms among Chinese adolescents has continued to rise, with the increase being particularly pronounced among girls. As risk factors have increased, the prevalence of psychological symptoms continues to trend (3). Research indicates that psychological symptoms during adolescence can adversely affect academic performance and peer relationships, and may even lead to serious self-harm or suicidal behavior, posing a serious threat to adolescent health (4). Research has also found that the negative effects of psychological symptoms during adolescence persist into adulthood, increasing the risk of developing mental health conditions such as depression and anxiety in adulthood and posing a threat to adult health (5). Research shows that family environmental factors have a significant impact on adolescents’ psychological symptoms, and that higher levels of parental educational attainment have a positive effect on healthy development (6). In addition, numerous studies have shown that factors such as dietary habits, sleep quality, physical fitness, and physical activity have a significant impact on psychological symptoms in adolescents (7, 8). However, a review of previous studies reveals that most research on the factors influencing psychological symptoms in adolescents has focused on the positive or negative effects of a single factor. In contrast, few studies have analyzed the joint effects of multiple factors. Previous research indicates that the factors influencing psychological symptoms in adolescents are multifaceted; therefore, it is necessary to conduct a comprehensive analysis of these factors across multiple dimensions to better develop intervention and guidance strategies (9).

Among the various factors influencing psychological symptoms in adolescents, the impact of community spatial and environmental factors on their mental health is receiving increasing attention and recognition. Community spatial and environmental factors encompass a range of aspects; for example, noise pollution in the surrounding community can affect adolescents’ sleep quality, leading to the onset of psychological symptoms (10). Research also shows that the level of green space in a community can affect adolescents’ mental health; this may be because the amount of green space is linked to adolescents’ physical activity levels, which in turn indirectly influence their psychological symptoms (11). In addition, the accessibility of transportation in the surrounding community can also affect adolescents’ psychological symptoms. Research shows that the accessibility of transportation in the surrounding community influences adolescents’ levels of physical activity, which in turn symptoms (12). At the same time, air quality in community spaces can also affect adolescents’ psychological symptoms. A longitudinal study of Chinese adolescents found that for every one-unit increase in PM2.5 levels, scores on the adolescent depression scale rose by 0.319 points, indicating a more severe tendency toward depression (13). This suggests a close association between air quality and psychological symptoms. Other studies have shown that air pollution can trigger neuroinflammatory responses, affecting brain structure and connectivity, thereby disrupting emotional regulation and cognition (14). In addition, factors such as the artistic design and architectural quality of community spaces can indirectly influence adolescents’ mental health, thereby having a direct or indirect impact on their well-being (15). Previous studies have addressed the issue of inconsistent standards for evaluating community spaces. Many of these studies have employed self-assessment questionnaires to conduct both subjective and objective evaluations, yielding reliable results (16, 17). Subjective satisfaction with the community environment is a key factor influencing adolescents’ well-being, quality of life, and mental health. A satisfying physical environment (e.g., safety, hygiene, and facilities) and social environment (e.g., social interactions, community cohesion) can significantly enhance adolescents’ subjective well-being, life satisfaction, and mental health (18). This suggests that environmental factors in community spaces have a significant impact on adolescents’ mental health; a more thorough analysis of these factors will play a positive role in promoting adolescents’ well-being and provide valuable support.

Among the various factors influencing psychological symptoms in adolescents, family environment also plays a role in their development. Research indicates that parenting styles have a significant impact on adolescents’ psychological symptoms; a harmonious and democratic family environment promotes their mental health, whereas authoritarian, authoritarian-authoritative, overprotective, or permissive parenting styles on the development of psychological problems in adolescents (19). In addition, research has shown that there is a close link between parents’ educational attainment and adolescents’ mental health; parents with higher levels of education pay closer attention to changes in their children’s mental health and are more willing to listen to their children’s emotional concerns, thereby providing better guidance and support (20). Conversely, families where parents have lower levels of education often neglect their children’s education or place too much emphasis on academic performance while overlooking changes in their mental state, which has a serious negative impact on adolescents’ mental health (21). In addition, there is a certain correlation between parents’ educational attainment and their approach to raising children. Parents with higher levels of education not only emphasize developing good habits and physical exercise in their children but also place greater emphasis on their children’s mental health. They pay close attention to and take seriously any unusual behaviors or psychological changes that arise, enabling them to promptly address or resolve potential psychological issues and thereby better prevent the onset of psychological symptoms (22). It is worth noting that community space, environmental satisfaction, and parents’ educational attainment—key dimensions of the community environment and family education that shape adolescents’ development—significantly influence adolescents’ psychological symptoms. These two factors represent the primary external influences on adolescents outside of formal schooling (23). However, there has been relatively little research to date on the relationship between Chinese parents’ educational attainment and adolescents’ psychological symptoms, and even fewer studies have utilized nationally representative samples.

A review of the literature reveals that multiple factors influence adolescents’ psychological symptoms, with the community spatial environment and parents’ educational attainment exerting significant effects. However, previous studies have primarily focused on the association between individual factors and adolescents’ psychological symptoms, while research on the joint effects of these two factors has been limited. Furthermore, previous studies have utilized relatively small sample sizes, limiting their representativeness.

Although previous studies have separately documented the associations of community environment and parental education with adolescent psychological symptoms, the theoretical and empirical basis for examining their joint effects warrants further elaboration. From the perspective of Bronfenbrenner’s ecological systems theory, adolescent development is shaped by the interplay of multiple environmental layers—the community (exosystem) and the family (microsystem)—rather than by isolated factors acting independently (23). The community spatial environment constitutes a critical external context that influences adolescents’ daily physical activities, social interactions, safety perceptions, and exposure to environmental stressors such as noise and air pollution (10). Concurrently, parents’ educational attainment serves as a key indicator of family socioeconomic resources, parenting practices, and health literacy, which collectively shape the quality of the proximal developmental environment (22). Importantly, these two contextual layers are not merely additive in their effects; they are likely to interact in meaningful ways.

Therefore, this study employs a nationally representative sample from China to analyze the independent and joint associations between the community spatial environment and parents’ educational attainment and psychological symptoms, thereby providing references and insights for the prevention and intervention of psychological symptoms in adolescents.

## 2 Methods

### 2.1 Participants

**Figure 1:**
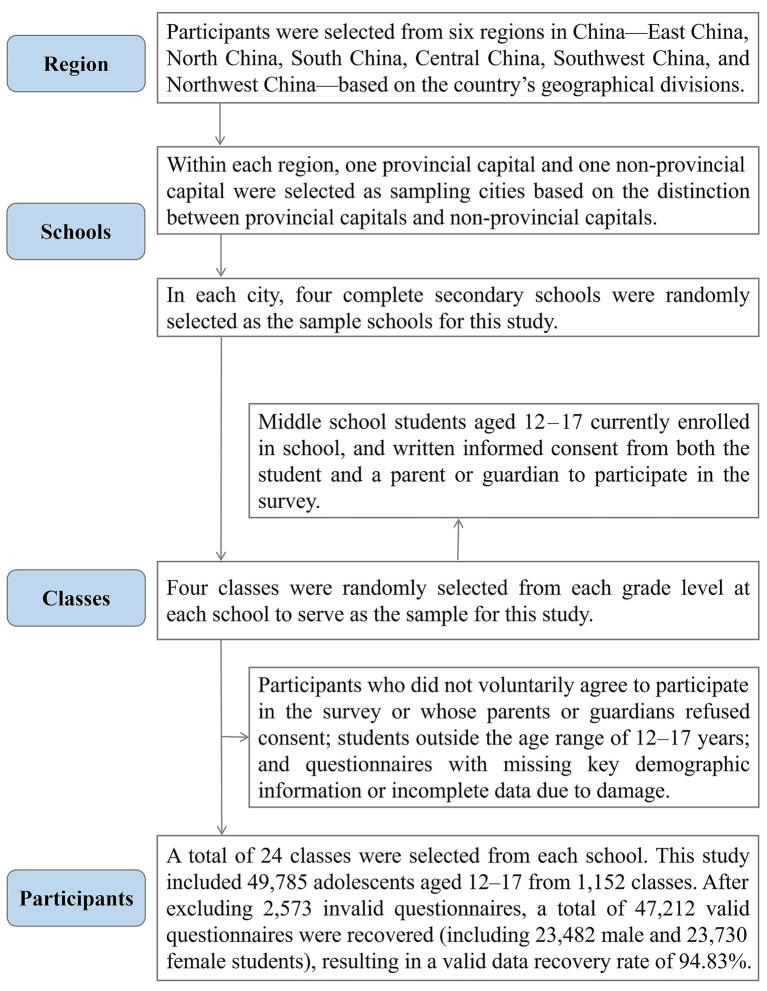
Sampling process for Chinese youth participants.

This study employed a three-stage stratified cluster sampling method to select participants. First, participants were selected from six regions in China—East China (Jinan, Yantai), North China (Shijiazhuang, Baoding), South China (Guangzhou, Huizhou), Central China (Wuhan, Yichang), Southwest China (Chengdu, Deyang), and Northwest China (Xi’an, Baoji)—based on the country’s geographical divisions. Second, within each region, one provincial capital and one non-provincial capital were selected as sampling cities based on the distinction between provincial capitals and non-provincial capitals. Non-provincial capital cities are selected from level within the province. Third, in each city, four complete secondary schools were randomly selected as the sample schools for this study. Fourth, four classes were randomly selected from each grade level at each school to serve as the study. A total of 24 classes were selected from each school. This study included 49,785 adolescents aged 12–17 from 1,152 classes. After excluding 2,573 invalid questionnaires, a total of 47,212 valid questionnaires were recovered (including 23,482 male and 23,730 female students), resulting in a valid data recovery rate of 94.83%. An *a priori* power analysis using G*Power 3.1 (OR = 1.5, *a* = 0.05, power = 0.95, df = 20) indicated that a minimum of 1,122 participants were required. Our final sample of 47,212 far exceeded this requirement, ensuring robust statistical power. Notably, the national representativeness of our sample was guaranteed by the three-stage stratified cluster sampling design across China’s six geographical regions, rather than by sample size alone. This design ensured broad coverage of adolescents from diverse regional and urban–rural backgrounds. The inclusion criteria for participants in this study were: middle school students aged 12–17, currently enrolled in school, and written informed consent from both the student and a parent or guardian to participate in the survey. The exclusion criteria for this study were: participants who did not voluntarily agree to participate in the survey or whose parents or guardians refused consent; students outside the age range of 12–17 years; and questionnaires with missing key demographic information or incomplete data due to damage. The sampling process for participants in this study is shown in Figure 1.

This study was conducted in accordance with the principles of the Declaration of Helsinki. The assessment was conducted after obtaining written informed consent from the parent/guardian and the participant, and after signing a written informed consent form. Approved by the Human Ethics Committee of Anqing Normal University (AQNU2023033).

### 2.2 Psychological symptom evaluation

In this study, the “Brief Inventory of Adolescent Psychological Health” was used to assess psychological symptoms in adolescents (24). This questionnaire consists of 15 specific items that reflect mental health status. It primarily assesses participants’ specific mental health status over the past 6 months. Each item is divided into six duration categories based on time the condition has persisted, ranging from less than 1 week to 6 months or longer. Participants should select the appropriate item based on their experience. A score of 1 is recorded for symptoms lasting 1 month or longer, and a score of 0 is recorded for symptoms lasting less than 1 month. The questionnaire is divided into three dimensions: emotional problems, behavioral problems, and social adjustment difficulties. A positive result is indicated when the scores for these three dimensions are ≥4, ≥1, and ≥2, respectively. A total score of ≥7 indicates the presence of psychological symptoms.

### 2.3 Community space environmental evaluation

Community space environmental evaluation refers to the overall characteristics of the community space environment, as reflected in adolescents’ subjective assessments and objective experiences. The questionnaire consisted of 10 items: “Accessibility by bus or subway,” “Community air quality,” “Community safety,” “Natural lighting and ventilation in the home,” “Building construction quality,” “Community living environment,” “Stairwell and elevator design,” “Community greening,” “Community landscape architecture,” and “Noise pollution.”

The selection of these 10 items was guided by a comprehensive review of established environmental assessment instruments and empirical evidence. Specifically, the item content was informed by validated scales including the Neighborhood Environment Walkability Scale for Youth (NEWS-Y) and its Chinese adaptation for children (NEWS-CC), the Residential Environmental Satisfaction Scale (RESS), and the ALPHA environmental questionnaire. These 10 items were purposively selected to capture the multi-dimensional nature of the community spatial environment that previous research has identified as relevant to adolescent mental health, encompassing: (a) accessibility (public transportation access); (b) environmental quality (air quality, noise pollution); (c) physical infrastructure (building quality, stairwell/elevator design, natural lighting and ventilation); (d) safety (community safety); and (e) aesthetics and green space (community greening, landscape architecture, living environment). Moreover, each of these items has been individually linked to adolescent psychological outcomes in prior studies (10–15), providing empirical justification for their inclusion.

For each item, participants rated their satisfaction on a 1-to-5 scale, ranging from “Very Dissatisfied” to “Very Satisfied,” based on their actual circumstances (25). Higher scores indicate greater satisfaction with the community space environment, with a total questionnaire score ranging from 10 to 50 points. Based on the percentile distribution of final scores, and in accordance with international standards, the P25 and P75 are used as cutoff points to classify responses into “Not satisfied,” “Generally satisfied,” and “Satisfied,” corresponding to scores of 10–29, 30–39, and 40–50, respectively (26–28). This questionnaire demonstrates good reliability and validity for assessing the community space environment among Chinese adolescents, with a Cronbach’s alpha coefficient of 0.86 (29).

### 2.4 Parents’ educational attainment evaluation

In this study, parents’ educational attainment was assessed using a self-report questionnaire. Participants were asked to report the highest level of education completed by their father and mother separately, and the higher of the two was selected for analysis. This approach is consistent with established protocols, including the PhenX Toolkit’s child-reported parental education attainment measure and the National Longitudinal Study on Adolescent Health, and is widely used in adolescent health research as a reliable proxy for family socioeconomic status (30). In this study, parents’ educational attainment was categorized into three levels based on the standard Chinese educational classification system: “Junior high school and below,” “High school,” and “University degree or above.”

### 2.5 Covariates evaluation

Based on previous research literature, multiple factors influence psychological symptoms in adolescents. Taking into account the practical aspects of this study, the covariates included in this study are age, gender, body mass index, standing long jump, family income, and sleep duration (31–33). Body mass index is calculated based on height and weight. Height, weight, and standing long jump are assessed using the testing methods and instruments specified in the National Student Physical Fitness Survey. Height is measured to the nearest 0.1 centimeter, weight to the nearest 0.1 kg, and standing long jump to the nearest 1 centimeter. In this study, family income and sleep duration were assessed using questionnaires completed by the participants themselves. Family financial income is categorized as <3,000 yuan/month,” “3,001–5,000 yuan/month,” “5,001–7,000 yuan/month,” and “>7,000 yuan/month.” Sleep duration is categorized as “<7 h/day,” “7–8 h/day,” and “≥8 h/day.”

### 2.6 Statistical analysis

In this study, categorical variables are presented as percentages, and comparisons between groups were performed using the chi-square test. Continuous variables are presented as means and standard deviations, and comparisons between groups were performed using *t*-tests. The association between community space environment and parents’ educational attainment and psychological symptoms was analyzed using binary logistic regression. Hierarchical regression analysis was conducted with the presence of psychological symptoms in adolescents as the dependent variable and the community space environment and parents’ educational attainment as independent variables. Model 1 is the crude model; Model 2 adjusts for age and BMI based on Model 1; Model 3 adjusts for standing long jump, family financial income, and sleep duration based on Model 2. This was done to further analyze the joint association between community space environment and parents’ educational attainment and psychological symptoms. This study used the presence of psychological symptoms in adolescents as the dependent variable and various combinations of, and conducted a binary logistic regression analysis using generalized linear models. The models were adjusted for age, BMI, standing long jump, family financial income, and sleep duration. An analysis of interaction effects was then conducted. After treating “Parents’ educational attainment” and “Community space environmental satisfaction” as dummy variables, an interaction analysis was conducted with psychological symptoms as the dependent variable. Data processing and analysis were performed using SPSS 25.0 software. A *p* < 0.05 was set as the threshold for two-sided statistical significance.

## 3 Results

This study conducted a cross-sectional assessment of community space environment, parents’ educational attainment, and psychological symptoms among 47,212 adolescents aged 12–17 years nationwide in China. The results showed that the prevalence of psychological symptoms among Chinese adolescents was 21.2%. The prevalence was higher among boys (22.1%) than among girls (20.4%), and the difference was statistically significant (*χ*^2^ = 21.055, *p* < 0.001). Only 33.5% of participants reported being “Satisfied” with their community space environment, and the genders was statistically significant (*χ*^2^ = 516.122, *p* < 0.001). Regarding parents’ educational attainment, the difference between genders was not statistically significant (*p* > 0.05). Comparison results for other indicators are shown in Table 1.

**Table 1:** A sex-specific comparison of the basic characteristics of Chinese youth.

Category	Boys	Girls	Total	*χ*^2^/*t*-value	*p*-value
Number	23,482	23,730	47,212		
Age (years)	14.64 ± 1.62	14.7 ± 1.65	14.67 ± 1.64	4.194	<0.001
Height (cm)	169.23 ± 9.27	160.94 ± 6.48	165.09 ± 9.00	112.749	<0.001
Weight (kg)	58.83 ± 13.42	51.19 ± 9.3	55.03 ± 12.15	71.972	<0.001
Body mass index	20.40 ± 3.71	19.72 ± 3.18	20.06 ± 3.46	21.277	<0.001
Standing long jump	205.63 ± 32.15	168.1 ± 21.4	186.8 ± 33.16	149.449	<0.001
Family financial income				206.734	<0.001
<3,000 yuan/month	2,526(10.8)	2,752(11.6)	5,278(11.2)		
3,001–5,000 yuan/month	7,856(33.5)	9,006(38.0)	16,862(35.7)		
5,001–7,000 yuan/month	7,101(30.2)	7,114(30.0)	14,215(30.1)		
>7,000 yuan/month	5,999(25.5)	4,858(20.5)	10,857(23.0)		
Sleep duration				97.641	<0.001
<7 h/day	3,632(15.5)	3,909(16.5)	7,541(16.0)		
7–8 h/day	16,020(68.2)	16,709(70.4)	32,729(69.3)		
≥8 h/day	3,830(16.3)	3,112(13.1)	6,942(14.7)		
Community space environmental satisfaction				516.122	<0.001
Satisfied	6,871(29.3)	8,928(37.6)	15,799(33.5)		
Generally	12,537(53.4)	12,007(50.6)	24,544(52)		
Not satisfied	4,074(17.3)	2,795(11.8)	6,869(14.5)		
Parents’ educational attainment				4.153	0.125
Junior high school and below	11,008(46.9)	10,954(46.2)	21,962(46.5)		
High School	7,728(32.9)	8,017(33.8)	15,745(33.3)		
University degree or above	4,746(20.2)	4,759(20.1)	9,505(20.1)		
Emotional problems	6,542(27.9)	6,504(27.4)	13,046(27.6)	1.202	0.273
Behavioral problems	6,637(28.3)	5,965(25.1)	12,602(26.7)	58.989	<0.001
Social adjustment difficulties	4,326(18.4)	3,938(16.6)	8,264(17.5)	27.300	<0.001
Psychological symptoms	5,186(22.1)	4,831(20.4)	10,017(21.2)	21.055	<0.001

The results show a statistically significant difference in the prevalence of psychological symptoms among adolescents across levels of community space environmental satisfaction (*χ*^2^ = 277.599, *p* < 0.001). There is also a statistically significant difference in the prevalence of psychological symptoms among adolescents across different levels of parents’ educational attainment (*χ*^2^ = 48.130, *p* < 0.001). See Table 2 for comparison results regarding psychological symptoms across other dimensions.

**Table 2:** A one-way comparison of psychological symptoms among Chinese adolescents.

Category	Psychological symptoms [*N*(%)]		*χ*^2^/*t*-value	*P*-value
	No	Yes		
Number	37,195	10,017		
Age (years)	14.67 ± 1.65	14.67 ± 1.59	0.082	0.935
Height (cm)	164.97 ± 8.99	165.42 ± 9.03	4.409	<0.001
Weight (kg)	54.73 ± 11.97	55.94 ± 12.76	8.829	<0.001
Body mass index	19.99 ± 3.41	20.32 ± 3.67	8.445	<0.001
Standing long jump	187.05 ± 32.97	185.7 ± 33.62	3.640	<0.001
Sex			21.055	<0.001
Boys	18,296(77.9)	5,186(22.1)		
Girls	18,899(79.6)	4,831(20.4)		
Family financial income			108.334	<0.001
<3,000 yuan/month	3,873(73.4)	1,405(26.6)		
3,001–5,000 yuan/month	13,406(79.5)	3,456(20.5)		
5,001–7,000 yuan/month	11,360(79.9)	2,855(20.1)		
>7,000 yuan/month	8,556(78.8)	2,301(21.2)		
Sleep duration			755.110	<0.001
<7 h/day	5,083(67.4)	2,458(32.6)		
7–8 h/day	26,253(80.2)	6,476(19.8)		
≥8 h/day	5,859(84.4)	1,083(15.6)		
Community space environmental satisfaction			277.599	<0.001
Satisfied	12,744(80.7)	3,055(19.3)		
Generally	19,556(79.7)	4,988(20.3)		
Not satisfied	4,895(71.3)	1974(28.7)		
Parents’ educational attainment			48.130	<0.001
Junior high school and below	17,020(77.5)	4,942(22.5)		
High School	12,494(79.4)	3,251(20.6)		
University degree or above	7,681(80.8)	1824(19.2)		

The results show that, overall, there were statistically significant differences among Chinese adolescents across the dimensions of emotional problems, behavioral problems, social adjustment difficulties, and psychological symptoms when comparing different levels of satisfaction with the community space environment (*χ*^2^ = 266.066, 286.485, 302.565, 277.599; *p* < 0.001); Similarly, statistically significant differences were found when comparing these dimensions based on parents’ educational attainment (*χ*^2^ = 27.327, 42.067, 74.356, 48.130; *p* < 0.001). The results of the comparisons stratified by gender are presented in Table 3.

**Table 3:** Single-factor comparison of community space environmental satisfaction, parents’ educational attainment, and psychological symptoms among Chinese adolescents.

Category	*N*	Emotional problems			Behavioral problems			Social adjustment difficulties			Psychological symptoms		
		*N* (%)	*χ*^2^-value	*P-*value	*N* (%)	*χ*^2^-value	*P*-value	*N* (%)	*χ*^2^-value	*P*-value	*N* (%)	*χ*^2^-value	*P*-value
Boys													
Community space environmental satisfaction			126.979	<0.001		115.692	<0.001		132.123	<0.001		117.801	<0.001
Satisfied	6,871	1821(26.5)			1823(26.5)			1,183(17.2)			1,425(20.7)		
Generally	12,537	3,293(26.3)			3,382(27.0)			2,134(17.0)			2,600(20.7)		
Not satisfied	4,074	1,428(35.1)			1,432(35.1)			1,009(24.8)			1,161(28.5)		
Parents’ educational attainment			10.489	0.005		14.465	0.001		49.433	<0.001		23.608	<0.001
Junior high school and below	11,008	3,157(28.7)			3,230(29.3)			2,211(20.1)			2,555(23.2)		
High School	7,728/	2,143(27.7)			2,150(27.8)			1,381(17.9)			1,695(21.9)		
University degree or above	4,746	1,242(26.2)			1,257(26.5)			734(15.5)			936(19.7)		
Girls													
Community space environmental satisfaction			146.232	<0.001		157.511	<0.001		163.517	<0.001		157.306	<0.001
Satisfied	8,928	2,233(25.0)			2057(23.0)			1,349(15.1)			1,630(18.3)		
Generally	12,007	3,247(27.0)			2,940(24.5)			1890(15.7)			2,388(19.9)		
Not satisfied	2,795	1,024(36.6)			968(34.6)			699(25.0)			813(29.1)		
Parents’ educational attainment			19.323	<0.001		28.866	<0.001		27.178	<0.001		26.800	<0.001
Junior high school and below	10,954	3,152(28.8)			2,924(26.7)			1963(17.9)			2,387(21.8)		
High School	8,017	2,115(26.4)			1947(24.3)			1,263(15.8)			1,556(19.4)		
University degree or above	4,759	1,237(26.0)			1,094(23.0)			712(15.0)			888(18.7)		
Total													
Community space environmental satisfaction			266.066	<0.001		286.485	<0.001		302.565	<0.001		277.599	<0.001
Satisfied	15,799	4,054(25.7)			3,880(24.6)			2,532(16.0)			3,055(19.3)		
Generally	24,544	6,540(26.6)			6,322(25.8)			4,024(16.4)			4,988(20.3)		
Not satisfied	6,869	2,452(35.7)			2,400(34.9)			1708(24.9)			1974(28.7)		
Parents’ educational attainment			27.327	<0.001		42.067	<0.001		74.356	<0.001		48.130	<0.001
Junior high school and below	21,962	6,309(28.7)			6,154(28.0)			4,174(19.0)			4,942(22.5)		
High School	15,745	4,258(27.0)			4,097(26.0)			2,644(16.8)			3,251(20.6)		
University degree or above	9,505	2,479(26.1)			2,351(24.7)			1,446(15.2)			1824(19.2)		

A sex-stratified binary logistic regression analysis was conducted, with the presence of psychological symptoms among Chinese adolescents as the dependent variable and community space environmental satisfaction and parents’ educational attainment as independent variables. Model 1 was the crude model; Model 2 adjusted for age and BMI; and Model 3 adjusted for standing long jump, family income, and sleep duration, based on Model 2. Overall results indicate that, compared to adolescents in the “Satisfied” group regarding community space environmental satisfaction, those in the “Not satisfied” group had a higher risk of developing psychological symptoms (OR = 1.65, 95% CI: 1.55–1.77) (*p* < 0.001). The results after stratification by gender are shown in Table 4.

**Table 4:** An analysis of the independent associations between Chinese adolescents’ satisfaction with community spaces and parents’ educational attainment and psychological symptoms.

Sex/variable	Grouping	Psychological symptoms					
		Model 1		Model 2		Model 3	
		OR (95% CI)	*P-*value	OR (95% CI)	*P-*value	OR (95% CI)	*P-*value
Boys							
Community space environmental satisfaction	Satisfied	1.00		1.00		1.00	
	Generally	1.00(0.93 ~ 1.08)	0.999	1.00(0.93 ~ 1.08)	0.981	1.01(0.94 ~ 1.09)	0.800
	Not satisfied	1.52(1.39 ~ 1.67)	<0.001	1.52(1.39 ~ 1.67)	<0.001	1.48(1.35 ~ 1.62)	<0.001
Parents’ educational attainment	University degree or above	1.00		1.00		1.00	
	High School	1.14(1.05 ~ 1.25)	0.003	1.16(1.06 ~ 1.26)	0.002	1.16(1.06 ~ 1.27)	0.002
	Junior high school and below	1.23(1.13 ~ 1.34)	<0.001	1.25(1.15 ~ 1.36)	<0.001	1.28(1.16 ~ 1.40)	<0.001
Girls							
Community space environmental satisfaction	Satisfied	1.00		1.00		1.00	
	Generally	1.11(1.04 ~ 1.19)	0.003	1.11(1.04 ~ 1.19)	0.003	1.10(1.03 ~ 1.18)	0.007
	Not satisfied	1.84(1.67 ~ 2.03)	<0.001	1.86(1.68 ~ 2.05)	<0.001	1.80(1.63 ~ 1.98)	<0.001
Parents’ educational attainment	University degree or above	1.00		1.00		1.00	
	High School	1.05(0.96 ~ 1.15)	0.298	1.00(0.93 ~ 1.07)	0.952	1.05(0.95 ~ 1.15)	0.356
	Junior high school and below	1.22(1.12 ~ 1.32)	<0.001	1.05(0.95 ~ 1.15)	0.34	1.23(1.12 ~ 1.35)	<0.001
Total							
Community space environmental satisfaction	Satisfied	1.00		1.00		1.00	
	Generally	1.06(1.01 ~ 1.12)	0.016	1.07(1.02 ~ 1.12)	0.010	1.07(1.02 ~ 1.13)	0.010
	Not satisfied	1.68(1.58 ~ 1.80)	<0.001	1.70(1.59 ~ 1.82)	<0.001	1.65(1.55 ~ 1.77)	<0.001
Parents’ educational attainment	University degree or above	1.00		1.00		1.00	
	High School	1.10(1.03 ~ 1.17)	0.005	1.10(1.03 ~ 1.17)	0.003	1.00(0.93 ~ 1.07)	0.952
	Junior high school and below	1.22(1.15 ~ 1.30)	<0.001	1.24(1.17 ~ 1.32)	<0.001	1.10(1.03 ~ 1.18)	0.003

To further analyze the joint relationship between community space environmental satisfaction and parents’ educational attainment with psychological symptoms, this study conducted a binary logistic regression analysis using a generalized linear model. The dependent variable was the presence of psychological symptoms among Chinese adolescents, while the independent variables were the various combinations of community space, environmental satisfaction, and parents’ educational attainment. Overall analysis results show that, with adolescents in the group reporting “Satisfied” with community space environmental satisfaction and “University degree or above” in parents’ educational attainment serving as the reference group, adolescents in the group reporting “Not satisfied” with community space environmental satisfaction and “Junior high school and below” in parents’ educational attainment had the highest risk of developing psychological symptoms (OR = 2.04, 95% CI: 1.81–2.30) (*p* < 0.001). Results stratified by gender are presented in Table 5.

**Table 5:** An analysis of the joint effects of Chinese adolescents’ satisfaction with community spaces and parents’ educational attainment on psychological symptoms.

Sex	Community space environmental satisfaction	Parents’ educational attainment	Psychological symptoms	
			OR (95% CI)	*P*-value
Boys	Satisfied	University degree or above	1.00	
		High School	1.10 (0.93 ~ 1.30)	0.283
		Junior high school and below	1.15 (0.98 ~ 1.35)	0.081
	Generally	University degree or above	0.98 (0.83 ~ 1.17)	0.855
		High School	1.06 (0.91 ~ 1.24)	0.468
		Junior high school and below	1.19 (1.02 ~ 1.38)	0.024
	Not satisfied	University degree or above	1.22 (1.00 ~ 1.50)	0.050
		High School	1.76 (1.48 ~ 2.11)	<0.001
		Junior high school and below	1.93 (1.63 ~ 2.29)	<0.001
Girls	Satisfied	University degree or above	1.00	
		High School	0.98 (0.84 ~ 1.15)	0.824
		Junior high school and below	1.15 (1.00 ~ 1.33)	0.051
	Generally	University degree or above	0.99 (0.84 ~ 1.16)	0.880
		High School	1.11 (0.96 ~ 1.28)	0.158
		Junior high school and below	1.32 (1.15 ~ 1.51)	<0.001
	Not satisfied	University degree or above	1.82 (1.48 ~ 2.25)	<0.001
		High School	1.88 (1.56 ~ 2.25)	<0.001
		Junior high school and below	2.10 (1.76 ~ 2.49)	<0.001
Total	Satisfied	University degree or above	1.00	
		High School	1.04 (0.92 ~ 1.16)	0.552
		Junior high school and below	1.16 (1.04 ~ 1.29)	0.007
	Generally	University degree or above	1.00 (0.89 ~ 1.12)	0.975
		High School	1.10 (0.99 ~ 1.22)	0.092
		Junior high school and below	1.26 (1.14 ~ 1.40)	<0.001
	Not satisfied	University degree or above	1.47 (1.27 ~ 1.69)	<0.001
		High School	1.85 (1.63 ~ 2.09)	<0.001
		Junior high school and below	2.04 (1.81 ~ 2.30)	<0.001

**Figure 2:**
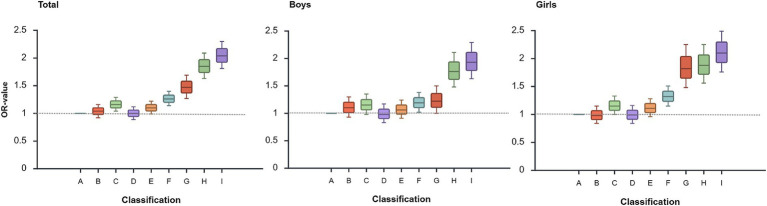
Trends in OR values for the joint effects of community space environmental satisfaction and parents’ educational attainment on psychological symptoms among Chinese adolescents.

As shown in Figure 2, as satisfaction with the community space environment increases and parents’ educational attainment decreases, the overall risk of adolescents developing psychological symptoms tends to rise, as indicated by the increasing OR values. This trend holds for both boys and girls.

**Table:** 

Community space environmental satisfaction	Parents’ educational attainment	Category
Satisfied	University degree or above	A
	High School	B
	Junior high school and below	C
Generally	University degree or above	D
	High School	E
	Junior high school and below	F
Not satisfied	University degree or above	G
	High School	H
	Junior high school and below	I

After treating “Parents’ educational attainment” and “Community space environmental satisfaction” as dummy variables, an interaction analysis was conducted with psychological symptoms as the dependent variable. The results showed that, compared with the reference group, the interaction term “Parents’ educational attainment (Junior high school and below) × Community space environmental satisfaction (Not satisfied)” exhibited an additional positive synergistic effect; that is, dissatisfaction with the community space environment amplifies the impact of psychological symptoms among adolescents in families with parents of low educational attainment (*p* < 0.05). Overall, the results indicate that the impact of parental educational attainment on psychological symptoms depends on satisfaction with the community space environment; the positive risk effect associated with low or moderate parental educational attainment is significantly amplified only when adolescents are dissatisfied with the community. Specific results are presented in Table 6.

**Table 6:** Analysis of the interaction effects of community space environmental satisfaction, parents’ educational attainment, and psychological symptoms among Chinese adolescents.

	Coefficient	SE	Wald *χ*^2^	*df*	*P*-value	OR	95% CI	
Constant	−1.51	0.046	1084.401	1	<0.001	0.221		
Parents’ educational attainment (Junior high school and below)	0.146	0.054	7.292	1	0.007	1.157	1.041	1.287
Parents’ educational attainment (High School)	0.035	0.058	0.353	1	0.552	1.035	0.924	1.16
Community space environmental satisfaction (Generally)	−0.002	0.06	0.001	1	0.975	0.998	0.888	1.122
Community space environmental satisfaction (Not satisfied)	0.382	0.073	27.154	1	<0.001	1.466	1.269	1.693
Parents’ educational attainment (Junior high school and below) × Community space environmental satisfaction (Generally)	0.088	0.07	1.597	1	0.206	1.092	0.952	1.253
Parents’ educational attainment (Junior high school and below) × Community space environmental satisfaction (Not satisfied)	0.183	0.089	4.251	1	0.039	1.201	1.009	1.429
Parents’ educational attainment (High School) × Community space environmental satisfaction (Generally)	0.058	0.075	0.591	1	0.442	1.059	0.915	1.227
Parents’ educational attainment (High School) × Community space environmental satisfaction (Not satisfied)	0.195	0.093	4.396	1	0.036	1.216	1.013	1.459

Control group, Parents’ educational attainment (University degree or above) × Community space environmental satisfaction (Satisfied).

## 4 Discussion

### 4.1 Prevalence of psychological symptoms and gender differences

To our knowledge, this study is the first to use a nationwide sample in China to analyze the independent and joint associations between adolescents’ satisfaction with their community space environment and their parents’ educational attainment, on the one hand, and psychological symptoms, on the other. The results of this study indicate that the prevalence of psychological symptoms among Chinese adolescents is 21.2%, which is higher than the 20.3% reported by Zhang et al. (34) in their survey of Chinese adolescents. At the same time, the findings of this study are higher than the prevalence rate of mental disorders among adolescents aged 15–19 reported in global surveys (13.96%) (35). This indicates that the prevalence of psychological symptoms among Chinese adolescents poses a significant challenge, warranting sufficient attention and priority. Consequently, effectively analyzing the multifaceted factors that influence the occurrence of psychological symptoms in adolescents is both important and practical. However, our prevalence estimate differs from some previous reports. However. The prevalence of psychological symptoms in this study was lower than the 26.3% reported by Li et al. (36) in their survey of Chinese adolescents. There are multiple factors contributing to the discrepancies in research findings. First, differences in the assessment tools used across studies may lead to varying results. Second, regional differences in the study populations may also be a significant factor contributing to these discrepancies. Research has found that the prevalence of psychological symptoms among Chinese adolescents varies significantly by region, with higher rates observed in western regions compared to eastern regions. The primary factors contributing to these differences are variations in economic development, academic pressure, dietary habits, and family circumstances (37). The results of this study also show that the prevalence of psychological symptoms among adolescent boys in China is higher than that among girls. This finding differs from the conclusions of many other studies (38, 39). This discrepancy may be partly attributable to gender differences in symptom manifestation (boys tend to exhibit externalizing behaviors while girls show internalizing symptoms), lower help-seeking tendencies among boys, and potentially higher susceptibility to lifestyle-related risk factors, although culturally shaped gender role expectations may also play a role—yet direct evidence for adolescents aged 12–17 remains limited. Given the cross-sectional nature of our study, these interpretations remain speculative, and future longitudinal research with gender-specific analytical approaches is warranted to elucidate the underlying mechanisms.

### 4.2 Independent associations of community space environmental satisfaction and parents’ educational attainment with psychological symptoms

Among the various factors influencing psychological symptoms in adolescents, there is a close association between the community space environment and these symptoms. The results of this study indicate that there is a negative correlation between Chinese adolescents’ satisfaction with the community space environment and the prevalence of psychological symptoms; that is, the higher the satisfaction with the community space environment, the lower the prevalence of psychological symptoms. There are multiple reasons for this finding. First, the accessibility of public transportation or subways in residential areas, as a key indicator of satisfaction with the community space environment, is closely associated with adolescents’ psychological symptoms. Access to public transportation or subways is closely linked to levels of physical activity, which in turn indirectly influence adolescents’ psychological symptoms (40). Second, there is a strong correlation between adolescents’ community living environment and psychological symptoms. Studies have found that adolescents living in communities characterized by social disorder, widespread damage to public facilities, frequent violent incidents, and high crime rates—even if they have not been directly subjected to violence—experience significantly higher levels of psychological distress, leading to an increased prevalence of psychological symptoms (41, 42). Third, the noise environment in residential communities can also have a negative impact on the development of psychological symptoms in adolescents. Previous studies have found that higher levels of daytime and nighttime traffic noise in the home environment are significantly associated with higher levels of reported anxiety symptoms among adolescents (43). This demonstrates the close link between noisy environments and the presence of psychological symptoms. Other studies have found that, compared to peers living in more favorable environments, adolescents residing in highly disadvantaged neighborhoods at baseline experienced a significant reduction in sleep duration 2 years later—a decrease of 2.65 min per night. This reduction in sleep is associated with mood fluctuations and cognitive decline, and is closely linked to the development of psychological symptoms (44). Finally, the greening of the community environment and air quality are also significantly associated with psychological symptoms in adolescents. A study of 7,555 adolescents in London found that increased exposure to ozone was associated with a slower rate of improvement in executive function, while nitrogen dioxide and particulate matter also had negative effects on cognitive abilities, thereby influencing the occurrence of psychological symptoms (45). This suggests that various factors related to the community living environment are significantly associated with psychological symptoms in adolescents; therefore, future research should focus on the impact of these factors on adolescents’ psychological symptoms.

The findings of this study also indicate a significant association between parents’ educational attainment and adolescents’ psychological symptoms. There are multiple factors contributing to this result. First, parents’ educational attainment directly influences a family’s socioeconomic resources, including income, living conditions, and job stability, all of which are fundamental determinants of adolescents’ mental health (46). Conversely, lower levels of parental educational attainment are often associated with greater financial strain on the family; this strain can lead to family conflict and an unstable living environment, increasing emotional instability among adolescents and thereby contributing to the prevalence of psychological symptoms (47). Second, parents’ educational attainment significantly influences their educational beliefs, knowledge base, and ability to engage in their children’s education. A systematic review found a moderate negative correlation between parental involvement in education and depressive symptoms in adolescents (48). Conversely, parents with lower levels of education may face greater challenges in addressing their children’s complex emotional needs, monitoring their academic progress, and managing their behavior. This can lead to poor communication between parents and children, insufficient supervision, or the use of harsher disciplinary methods, all of which contribute to an increased prevalence of psychological symptoms among adolescents (49, 50). In addition, parents’ educational attainment is closely linked to their own mental health, which can indirectly influence their children’s mental health through a mediating effect. Previous research has found that lower levels of parental education are associated with higher levels of parental psychological distress, such as anxiety, depression, and stress, and these adverse changes can indirectly affect adolescents’ psychological well-being, leading to an increased prevalence of psychological symptoms (51). Finally, there is a strong correlation between parents’ educational attainment and the level of attention and concern they give to their children’s mental health. Research indicates that parents with higher levels of education may be better able to identify their children’s mental health concerns at an earlier stage; they are often able to take more timely and effective measures and employ more appropriate educational approaches to address mental health issues and promote healthy mental development (52). Conversely, parents with lower levels of education may mistake their children’s psychological symptoms for changes in personality or a phase of rebellious behavior, thereby delaying the opportunity for psychological intervention and leading to the onset of psychological symptoms (53).

### 4.3 Joint associations and interaction effects

We found a significant joint effect: adolescents with low parental education and community dissatisfaction had the highest risk of psychological symptoms (OR = 2.04, 95% CI: 1.81–2.30), substantially exceeding the independent effects of either factor. The synergistic interaction (*p* < 0.05) indicates that community dissatisfaction amplifies the risk associated with low parental education, and this amplification may stem from two mechanisms—differential vulnerability (low-education families lack resources to cope with environmental stressors) and the absence of community buffering effects that could otherwise compensate for family disadvantages. Theoretically, our findings support an ecological synergy model, demonstrating that family (microsystem) and community (exosystem) factors interact dynamically, and single-factor approaches may either overestimate or underestimate true effects by ignoring such contextual dependence. Practically, intervention strategies should prioritize high-risk neighborhoods with concentrated low-education families, integrate community environmental improvements with targeted parental health education, and adopt multi-level screening for adolescents facing dual adversities. Gender-specific patterns were suggestive but require further longitudinal investigation.

### 4.4 Limitations and future directions

To our knowledge, this study is the first to use a nationwide sample in China to analyze the independent and joint associations between satisfaction with the community space environment and parents’ educational attainment and adolescents’ psychological symptoms. This study may provide some reference and assistance for the prevention and intervention of adolescents’ psychological symptoms. However, this study also has certain limitations. First, as a cross-sectional study, it can only analyze the associations between variables and cannot determine causal relationships. Future studies should employ a prospective cohort design to better analyze causal relationships. Second, the factors influencing adolescents’ psychological symptoms are multifaceted; the limited number of covariates included in this study may introduce some bias into the results. Future studies should incorporate more covariates, such as physical activity and dietary habits, to better analyze the relationships among these variables. This study employed a self-assessment questionnaire to evaluate the community space environment, which may have introduced some bias in the results. Future studies should adopt more objective evaluation methods to improve the accuracy of the results. Finally, this study categorizes the evaluation of community space environments into “Not satisfied,” “Generally satisfied,” and “Satisfied,” using the P25 and P75 as thresholds. This approach may result in some variation in the analysis results; in future studies, continuous variables could be used for evaluation.

## 5 Conclusion

The prevalence of psychological symptoms among Chinese adolescents is relatively high, and there are both independent and joint associations between community space environmental satisfaction and parents’ educational attainment and these psychological symptoms. Future community planning should focus on greening and traffic safety, and at the same time strengthen health education for families with low education levels.

## Data Availability

The raw data supporting the conclusions of this article will be made available by the authors, without undue reservation.
